# Repeated semaglutide treatment attenuates cocaine-vs-food choice in male and female rats

**DOI:** 10.1038/s41386-026-02386-2

**Published:** 2026-04-15

**Authors:** Nicholas Heslep, Samuel A. Marsh, Matthew L. Banks

**Affiliations:** Department of Pharmacology and Toxicology, Virginia Commonwealth University School of Medicine, Richmond, VA USA

**Keywords:** Reward, Addiction

## Abstract

The present study determined the effectiveness of the glucagon-like peptide 1 agonist semaglutide to attenuate cocaine-vs-food choice in male and female rats. Repeated 5-day semaglutide treatment decreased cocaine choice and significantly reduced body weight. These preclinical results support the clinical evaluation of semaglutide as a candidate cocaine use disorder medication.

## Introduction

In addition to the ongoing opioid crisis, cocaine use disorder continues to present as a public health issue and for which there are currently no Food and Drug Administration-approved pharmacotherapies. This absence of effective medications for cocaine use disorder highlights the need for preclinical research to identify and evaluate candidate treatments. Towards that goal, Glucagon-like Peptide 1 (GLP-1) agonists have emerged as potential medications for substance use disorder based on both the neuroanatomical location of GLP-1 receptors and preliminary preclinical and human laboratory studies (for review, see [1]). For example, acute pretreatment with the GLP-1 agonist exenatide attenuated cocaine-induced locomotor activity and conditioned place preference [2–4]. Furthermore, acute activation of GLP-1 receptors either chemogenetically or pharmacologically attenuated cocaine-induced reinstatement [4, 5]. Moreover, acute pretreatment with the GLP-1 agonist exendin-4 or semaglutide attenuated cocaine self-administration in mice and rats [4, 6]. Acute pretreatment with a single dose of the GLP-1 agonist exenatide failed to attenuate cocaine self-administration and subjective effects in humans [7]. However, just as in the preclinical studies cited above, the authors of this human laboratory study acknowledged multiple experimental design limitations including only testing a single exenatide dose, and acute pharmacological treatments rather than repeated or sub-chronic treatments that have been shown to enhance predictive validity [8].

The goal of the present study was to determine the effectiveness of repeated 5-day semaglutide treatment on cocaine-vs-food choice in male and female rats using experimental designs demonstrated to enhance translational validity [8]. A cocaine choice procedure was utilized because the primary dependent measure “cocaine choice” allows for the simplified assessment of pharmacological treatments that are selective for addictive drug reinforcement vs. generalized alterations in operant responding due to cognitive or motor impairment [8]. In addition, preclinical drug choice procedures allow for the simplified assessment of the two primary clinical treatment goals which are to (1) attenuate addictive drug self-administration and (2) promote behavioral allocation away from the addictive drug and towards behaviors maintained by non-drug reinforcers such as family or work [8]. We hypothesized that if semaglutide warrants consideration as a candidate medication for cocaine use disorder, then repeated 5-day semaglutide treatment should attenuate cocaine-vs-food choice.

## Methods

Detailed methods are reported in supplemental materials. Animal research and maintenance were conducted following the 2011 NIH Guide for the Care and Use of Laboratory Animals, Eighth Edition. All enrichment and experimental protocols were approved by the Virginia Commonwealth University Institutional Animal Care and Use Committee.

## Results

**Fig. 1:**
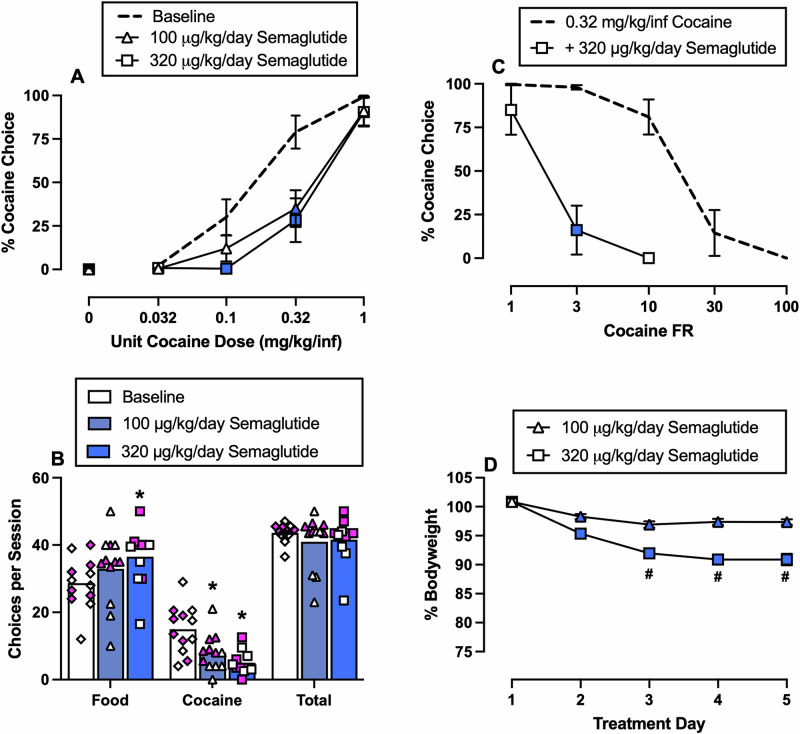
Effects of repeated 5-day semaglutide treatment on cocaine-vs-food choice in male and female rats. Panel **A** shows percent cocaine choice as a function of unit cocaine dose during each semaglutide treatment. Panel **B** shows session food, cocaine and total reinforcers. Panel **C** shows repeated 320 g/kg/day semaglutide treatment effects on 0.32 mg/kg/infusion cocaine choice under the behavioral economic-demand procedure. Panel **D** shows changes in bodyweight for each semaglutide dose over the five treatment days. Panels **A** and **B** show the mean±SEM of the last two days of each treatment for 12 rats (6 M/6 F). Panel **C** shows mean±SEM for 7 rats (4 M/3 F). Purple-filled symbols in Panel **B** denote individual female data points and open symbols denote individual male data points. Filled symbols or asterisks denote significance (*p* < 0.05) compared to baseline for Panels A and B. Panel **D** shows mean±SEM for 12 rats (6 M/6 F). ^#^ denotes significance compared to 100 g/kg/day semaglutide within a treatment day and filled symbols denote significance compared to Day 1 in Panel D.

Under the within-session cocaine choice dose-effect procedure, increasing cocaine doses maintained a dose-dependent increase in cocaine choice (Fig. 1A). Repeated 100 and 320 g/kg/day semaglutide significantly attenuated 0.32 mg/kg/infusion cocaine choice and 320 µg/kg/day semaglutide attenuated 0.1 mg/kg/infusion cocaine choice (Fig. 1A; semaglutide: F_1.5,19.3_ = 15.2, *p* = 0.0003; cocaine: F_1.3, 17.2_ = 85.8, *p* < 0.0001; interaction: F_3.3, 34.2_ = 3.7, *p* = 0.017). Additionally, 320 g/kg/day semaglutide increased session food choices and both 100 and 320 g/kg/day semaglutide decreased session cocaine choices without altering session total choices (Fig. 1B; dependent measure: F_1.1, 11.8_ = 105, *p* < 0.0001; interaction: F_1.7, 17.6_ = 8.4, *p* = 0.004). Under the cocaine choice behavioral-economic demand procedure, repeated 320 g/kg/day semaglutide treatment attenuated 0.32 mg/kg/infusion cocaine choice at FR3 (Fig. 1C; cocaine FR: F_1.9, 11.4_ = 13, *p* = 0.0013; semaglutide: F_1, 6_ = 35, *p* = 0.001; interaction: F_1.5, 5.3_ = 8.3, *p* = 0.027). Figure 1D shows that repeated 5-day 100 g/kg/day and 320 g/kg/day semaglutide decreased body weight (Day: F_1.5,20.4_ = 108.7, *p* < 0.0001; semaglutide dose: F_1, 14_ = 31.9, p < 0.0001; interaction: F_1, 6.4_ = 35.6, *p* = 0.0009). There were no significant sex differences for any dependent measure.

## Discussion

The present study determined the effectiveness of repeated 5-day semaglutide treatment on cocaine-vs-food choice in male and female rats. There were two main findings. First, both repeated 0.1 and 0.32 mg/kg/day semaglutide significantly attenuated cocaine choice and promoted behavioral reallocation away from cocaine and towards food. Second, 0.32 mg/kg/day semaglutide treatment decreased the cocaine FR value that rats reallocated their behavior away from cocaine and towards food under the behavioral economic-demand procedure. Under both procedures, semaglutide reductions in cocaine choice and reciprocal increases food choice were accompanied by decreased bodyweights suggesting that semaglutide effects on cocaine reinforcement were greater than its effects on food reinforcement. Overall, these preclinical results support the clinical evaluation of semaglutide as a candidate cocaine use disorder medication as suggested by others [1, 9, 10].

Surprisingly, semaglutide treatment effects on cocaine-vs-food choice were in the opposite direction that would be hypothesized for clinically used obesity treatments and pharmacological treatments that decrease body weight. For example, semaglutide treatment effects on cocaine choice were in contrast to lorcaserin treatment which failed to attenuate cocaine-vs-food choice in monkeys [11] and cocaine use in a double-blind placebo-controlled clinical trial despite body weight reductions [12]. The present results suggest that the mechanisms mediating GLP-1 agonists effects on cocaine self-administration are different than GLP-1 agonist mediated reductions in bodyweight. Although the underlying neurobiological mechanisms of semaglutide effects on body weight have been investigated [13], the mechanisms underlying semaglutide-induced reductions in cocaine choice remain to be fully elucidated because semaglutide does not cross the blood-brain barrier. However, GLP-1 receptors are expressed on ventral tegmental area (VTA) GABAergic neurons and chemogenetic activation of GLP-1 receptors decreased VTA dopaminergic activity [5]. Because cocaine reinforcement relies more on the mesolimbic dopamine pathway than food reinforcement, this may explain the selective semaglutide effects on cocaine-vs-food choice in the present study. Future directions should determine whether GLP-1 antagonists block semaglutide or other GLP-1 agonist effects on drug self-administration endpoints.

## Supplementary information

Supplemental Methods

## Data Availability

The datasets generated during and/or analyzed during the current experiments are available from the corresponding author on reasonable request.
