# Supplementary material for: Repeated semaglutide treatment attenuates cocaine-vs-food choice in male and female rats
Source: Neuropsychopharmacology. 2026 Apr 15;51(7):1144–6. doi: 10.1038/s41386-026-02386-2 (PMC13191343; doi:10.1038/s41386-026-02386-2)
Supplement: Supplementary file 1 — Supplemental Methods [file 41386_2026_2386_MOESM1_ESM.docx]

**SUPPLEMENTAL MATERIALS FOR**

**Repeated semaglutide treatment attenuates cocaine-vs-food choice**

**in male and female rats**

Nicholas Heslep^1^, Samuel A. Marsh^1^ Matthew L Banks^1,#^

^1^Department of Pharmacology and Toxicology, Virginia Commonwealth University School of Medicine, Richmond, VA, USA

Methods… 2

**Methods:**

Subjects: A total of 19 (8 Male, 11 Female) Sprague-Dawley rats were purchased from a commercial supplier (Inotiv, Frederick, MD, USA) and utilized across the two experiments. Nine rats participated in the within-session cocaine choice experiments, but not the cocaine choice behavioral economic demand experiments. Four rats participated in the cocaine choice behavioral-economic demand experiments, but not in the within-session cocaine choice experiments. Three rats participated in both the within-session cocaine choice and behavioral economic demand experiments. Weights at purchase were approximately 225-275g for females and 250-300g for males. All subjects were single-housed in temperature-controlled and AAALAC-accredited vivarium under a 12-h light:dark cycle running from 6 PM to 6 AM. Subjects were given *ad libitum* access to both food (Tekland Rat Diet, Envigo) and water in their home cage and were weighed weekly, except during repeated dosing experiments when subjects were weighed daily. Animal research and maintenance were conducted following the 2011 NIH Guide for the Care and Use of Laboratory Animals, Eighth Edition. All enrichment and experimental protocols were approved by the Virginia Commonwealth University Institutional Animal Care and Use Committee (IACUC).

Subjects were aseptically implanted with an indwelling intravenous (IV) jugular catheter and vascular access port as previously described [1]. Catheters were flushed daily with 0.1 mL of a sterile prophylactic mixture (cefazolin, 50 mg/mL; heparin, 250 U/mL in saline). Catheter patency was confirmed at the end of each experiment by administering IV methohexital (1.6 mg/0.1 mL) and catheters were considered patent upon observation of an instantaneous loss of muscle tone and ambulation. Only animals with patent catheters were included for data and graphical analysis.

Apparatus: Modular operant chambers (Med Associates, St. Albans, VT) housed in sound-attenuating chambers were used for all behavioral experiments as described previously [1]. Briefly, each retractable lever had tricolor LED lights (red, yellow, green) directly above. A Syringe pump (PHM-100, Med Associates) was connected to a fluid swivel (275/22PS, Instech Laboratories, Plymouth Meeting, PA), and the IV line was protected by a stainless steel, magnetic tether (Instech Laboratories). The operant chamber also contained a retractable dipper (Med Associates) with a 0.1 mL cup for liquid food delivery (32% vanilla-flavored Ensure^®^ diluted in tap water, Abbot Laboratories, Chicago, IL). All behavioral experiments were conducted using custom programs written in Med-State Notation (Med Associates) that are available upon request or previously published [1].

Procedures: Rats were trained to respond for both IV cocaine infusions and presentations of 32% vanilla-flavored Ensure (Abbott Laboratories, Chicago, IL) and then on the terminal cocaine-vs-food choice procedure using published methods [1]. Briefly, the terminal within-session cocaine-vs-food choice procedure consisted of five 20-min response components with a 5-min timeout between each component. During each timeout, subjects received a non-contingent infusion of the cocaine dose available and a non-contingent presentation of liquid food. Increasing cocaine doses (0, 0.032, 0.1, 0.32, 1.0 mg/kg/infusion) were available as the alternative to food during successive components under a concurrent fixed-ratio (FR)5:FR5 schedule of reinforcement. Cocaine dose was varied by changing the infusion duration (300 g: 0, 0.5, 1.56, 5, and 15.6 s of pump time during components 1-5, respectively). Subjects could complete up to 10 total ratio requirements across both levers, with responding on one lever resetting the ratio requirement for the other lever. Cocaine choice was considered stable when the smallest unit cocaine dose that maintained ≥ 80% choice in an individual subject did not vary more than 0.5 log units over three consecutive days.

Once cocaine choice was stable, subjects were administered 100 or 320 µg/kg/day semaglutide subcutaneously one hour before the choice session for five consecutive days (i.e., Monday – Friday). In addition, subjects were weighed before each daily semaglutide administration. Treatment weeks were separated by non-treatment weeks where subjects were still run on cocaine choice sessions but were not treated with semaglutide. Treatment weeks were only initiated if cocaine choice showed no significant difference compared to pretreatment levels.

For the cocaine choice demand procedure, after initial cocaine- and food-maintained training using our published methods [1], rats were trained under a concurrent FR5:FR5 schedule of cocaine and food during daily one-hour sessions. There was a 20-s timeout after each reinforcer presentation and there was no limit on the number of cocaine or food reinforcers earned. Once cocaine choice was stable defined as percent cocaine choice did not vary more than 20% of the three-day average, the cocaine FR was decreased to FR1 while holding the food FR constant at 5. Once choice behavior stabilized, the subsequent Monday was cocaine FR1, Tuesday was cocaine FR3, Wednesday was cocaine FR10, Thursday was cocaine FR32, and Friday was cocaine FR100. Once cocaine choice was eliminated at a given FR value, rats were returned to cocaine FR1 until cocaine choice returned. Baseline 0.32 mg/kg/infusion cocaine choice was determined in all rats before being treated with 320 µg/kg/day semaglutide subcutaneously one hour before the choice session and redetermining the cocaine choice behavioral economic demand function. During 320 μg/kg/day semaglutide treatment, rats continued to receive daily semaglutide treatments for all five days, and the cocaine FR remained constant for the remainder of that treatment week if a rat completely reallocated their behavior towards food and away from cocaine to assess potential tolerance to semaglutide treatment effects.

Drugs: Cocaine HCl was provided by the National Institute on Drug Abuse Drug Supply Program (Bethesda, MD). Semaglutide (HY-114118) was purchased from MedChemExpress (Monmouth Junction, NJ). Methohexital sodium (PAR Pharmaceutical, Chestnut Ridge, NJ) was purchased from a commercial supplier. All solutions were dissolved in bacteriostatic saline, and IV solutions were passed through a 0.22 µm sterile filter before use. Semaglutide solutions were made up fresh before each treatment week in amber vials that were wrapped in foil and stored under refrigeration. All drug doses were expressed as the salt or base forms listed above.

Statistical Analysis: The primary dependent measures were percent cocaine choice, defined as [(total number of cocaine reinforcers earned ÷ total number of cocaine and food reinforcers earned)* 100] and number of reinforcers (total, cocaine, and food) per session. The primary independent variables were semaglutide and cocaine dose or cocaine FR. Data from the last two days of each semaglutide treatment period were analyzed using a two-way repeated-measures ANOVA or mixed-effect analysis as appropriate, with cocaine or semaglutide dose as the main factors under the within-session cocaine choice dose-effect procedure. Raw daily body weights were individually normalized to the previous body weight before the first semaglutide dose for that week and expressed as a percentage. Post-hoc comparisons using a Dunnett’s or Sidak post-hoc test were conducted following a significant semaglutide treatment effect and/or interaction. The Geisser-Greenhouse correction was used for any sphericity violations. Statistical significance was established *a priori* at the 95% confidence level (p < 0.05).

**References**

1 Townsend EA, Schwienteck KL, Robinson HL, Lawson ST, Banks ML. A drug-vs-food “choice” self-administration procedure in rats to investigate pharmacological and environmental mechanisms of substance use disorders. J Neurosci Methods. 2021:109110.
